# Glutamate-associated research in Parkinson’s disease: a bibliometric analysis

**DOI:** 10.3389/fnagi.2025.1569924

**Published:** 2025-08-18

**Authors:** Yan-Jun Chen, Ming-Rong Xie, Sheng-Qiang Zhou, Fang Liu

**Affiliations:** ^1^Graduate School of Hunan University of Chinese Medicine, Changsha, China; ^2^National TCM Master Liu Zuyi Inheritance Studio, The Affiliated Hospital of Hunan Academy of Chinese Medicine, Changsha, China; ^3^The First Clinical College of Nanjing University of Chinese Medicine, Nanjing, China

**Keywords:** Parkinson’s disease, glutamate, alpha-synuclein, neuroinflammation, synaptic plasticity

## Abstract

**Background:**

Parkinson’s disease (PD) is a common neurodegenerative disease. As an important excitatory neurotransmitter, glutamate plays a key role in the nervous system. The imbalance of glutamate levels, the dysfunction of related receptors, and the abnormal activity of transporters are closely associated with the pathological process of PD. This study aims to explore the research hotspots, frontiers, and development directions of PD and glutamate.

**Method:**

The relevant publications were obtained from the Web of Science. CiteSpace, VOSviewer, and bibliometrix were used for visualization and quantitative evaluation.

**Results:**

A total of 2,488 publications related to the research topic were included. From 2001 to 2024, the annual number of publications showed a fluctuating upward trend. The United States was a leader in this field, working closely with China, Italy, the United Kingdom, and France. Karolinska Institute was the most active institution. Core journals were mainly related to neuroscience, covering basic research, clinical applications, and pharmacology. Dr. Conn, P. Jeffrey was the most productive author. The paper “*The functional anatomy of basal ganglia disorders*” was the most co-cited literature. High-frequency keywords included PD, glutamate, basal ganglia, oxidative stress, dopamine (DA), neurons, alpha-synuclein (a-Syn), glutamate receptors, and synaptic plasticity. In recent years, a-Syn and neuroinflammation were the research topics with strong burst power.

**Conclusion:**

Research on PD and glutamate focused on countries with increasing aging. The collaboration of different countries and institutions was conducive to promoting the development of this field. The research hotspots included basal ganglia, oxidative stress, DA, neurons, a-Syn, glutamate receptors, and synaptic plasticity. a-Syn and neuroinflammation may be research directions in the future.

## 1 Introduction

Parkinson’s disease (PD) is a neurodegenerative disease that commonly occurs in middle-aged and elderly people. The main pathological change of PD is the degeneration of dopaminergic neurons in the substantia nigra, resulting in decreased synthesis and release of dopamine (DA) in the central nervous system (Kalia and Lang, 2015). Although the medical community has made significant progress in the research of PD, its pathogenesis is still unclear.

Glutamate is a common excitatory neurotransmitter that plays a crucial role in maintaining the normal function of the nervous system (Zhou and Danbolt, 2014). In the nervous system, glutamate triggers the transmission of neural signals by binding to specific receptors and causing changes in the membrane potential of neurons (Traynelis et al., 2010). Glutamate is involved in normal neural conduction processes, regulates synaptic plasticity, and affects the strength of connections between neurons (Alix and Domingues, 2011). In addition, glutamate is widely expressed in the brain and plays an important role in cognitive function, learning, and memory (McEntee and Crook, 1993).

Related studies have shown that the imbalance of glutamate levels, related receptor dysfunction, and abnormal transporter activity are closely related to the pathological process of PD. Motor dysfunction of PD is strongly associated with increased glutamate levels in the basal ganglia (Nandhu et al., 2011). Mice lacking mGluR4 subtype of metabotropic glutamate receptors (mGluRs) show marked impairment in their ability to learn complex movements (Gerlai et al., 1998). Targeted treatment of glutamate receptors can alleviate motor symptoms of PD, protect neurons, and increase the anti-PD efficacy of dopaminergic drugs (Zhang et al., 2019). Glutamate transporters, such as excitatory amino acid transporters, are responsible for the uptake of glutamate from the synaptic cleft to neurons and glial cells to maintain glutamate homeostasis. In PD, the expression and function of glutamate transporters are significantly decreased, leading to the accumulation of glutamate in the synaptic cleft, which further aggravates excitotoxic damage and continuously promotes the progress of PD (Zhang et al., 2016).

Bibliometrics is a comprehensive knowledge system that focuses on quantitative analysis. It plays an important role in revealing the quantitative relationship of academic literature, exploring the dynamic characteristics of science and technology, and evaluating the research output. Using bibliometric methods to analyze the research related to glutamate in PD helps reveal research trends and hotspots, assess research impact, provide guidance for drug development and treatment, and promote interdisciplinary collaboration and communication.

## 2 Methods

### 2.1 Data retrieval

Web of Science (WoS) database covers the core literature resources of various disciplines, which can provide a comprehensive and representative data basis for bibliometric analysis. Data were obtained from the WoS core collection. Search using TS = Topic, Topic includes title, abstract, keyword plus, and author keywords. The search formula was ((TS = (glutamate)) OR TS = (glutamic acid)) AND TS = (Parkinson’s disease). The time was set before January 1, 2025. Inclusion criteria were as follows: (i) Publications related to glutamate and PD were included. (ii) The language type was limited to English. (iii) Only review and article were selected as publication types. Exclusion criteria were as follows: (i) Publications not related to glutamate and PD were excluded. (ii) Publication type excluded meeting abstract, editorial material, proceeding paper, letter, retracted publication, book chapters, early access, and biographical-item. (iii) Publications in languages other than English were excluded. Two researchers further analyzed the data and excluded duplicate literature. Finally, 2,488 publications were included.

### 2.2 Data analysis

CiteSpace helps researchers quickly identify research hotspots, frontier trends, and knowledge structures in a certain field by visualizing literature data (Chen, 2004). VOSviewer performs cluster analysis of literature, authors, institutions, etc., which clearly shows the relationships and differences between various research groups (van Eck and Waltman, 2010). Bibliometrix is a bibliometric analysis toolkit based on R language, which provides rich functions and methods for the comprehensive analysis of literature data (Blandini et al., 1996). Data analysis included the number of publications, country, institution, author, reference clustering, keyword co-occurrence, and burst. The data cover the period from 2001 to 2024. The time slice was 1 year, and the remaining Settings were set to default values. In cluster analysis, the modularity (Q) serves as a crucial indicator of community structure significance, with values exceeding 0.3 demonstrating robust community delineation. The silhouette (S) provides complementary validation of clustering quality, where scores above 0.7 indicate convincing and efficient partition patterns.

## 3 Results

### 3.1 Annual number of publications

**Figure 1:**
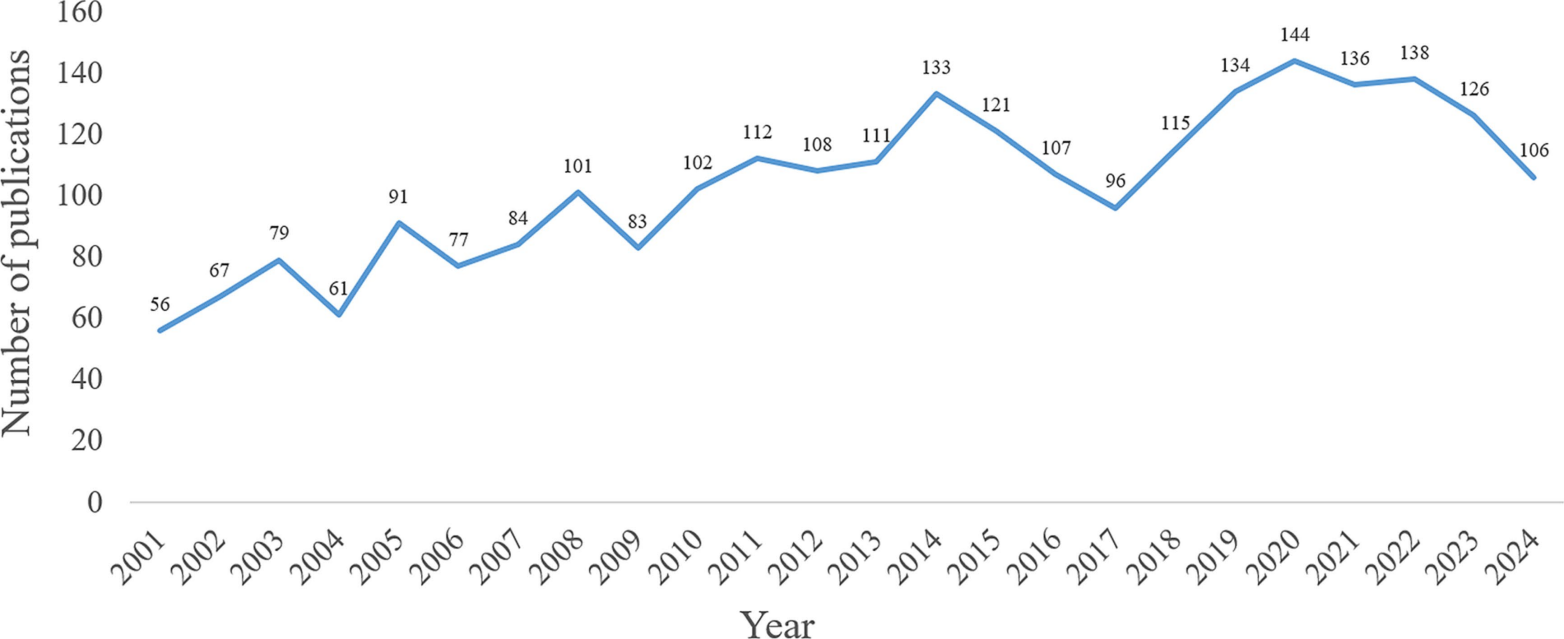
Annual number of publications from 2001 to 2024.

A total of 2,488 publications related to the research topic were obtained, which included 1837 articles and 651 reviews. From 2001 to 2024, the annual number of publications showed a fluctuating upward trend (Figure 1).

### 3.2 Country distribution

**Figure 2:**
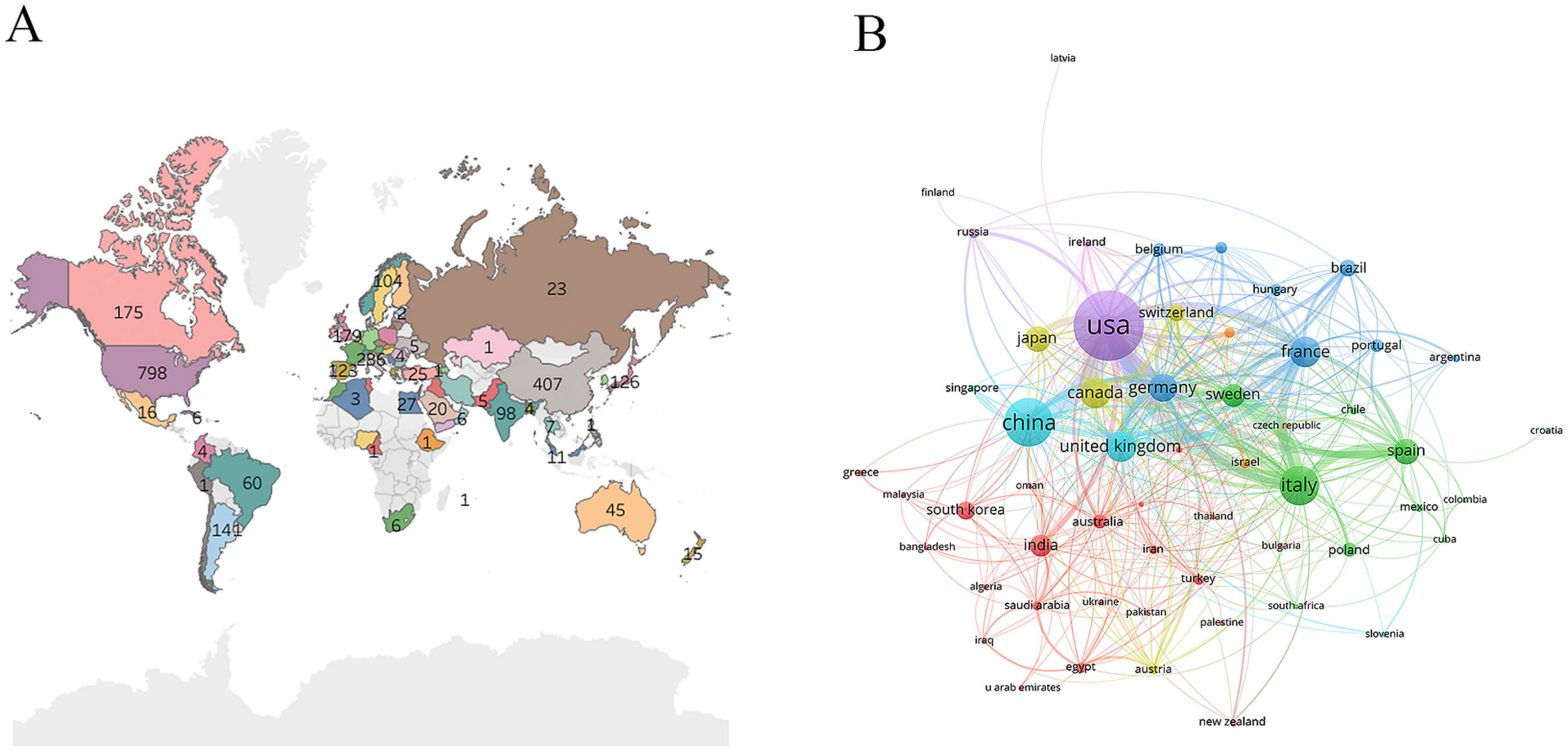
Analysis of countries. **(A)** Geographic distribution of countries. **(B)** Collaboration between countries.

The world map showed that active countries were concentrated in North America, Asia, and Europe (Figure 2A). The United States was a leader in this field, working closely with China, Italy, the United Kingdom, and France (Figure 2B). The United States was the most prolific country (798 publications), followed by China (407 publications) and Italy (286 publications) (Table 1).

**Table 1:** The top 10 productive countries.

Country	Number of publications	Citations
Usa	798	57,521
China	407	13,522
Italy	286	15,382
United Kingdom	179	11,225
Canada	175	9,765
France	173	9,967
Germany	157	9,310
Japan	126	5,232
Spain	123	5,026
Sweden	104	5,787

### 3.3 Research institutions

**Figure 3:**
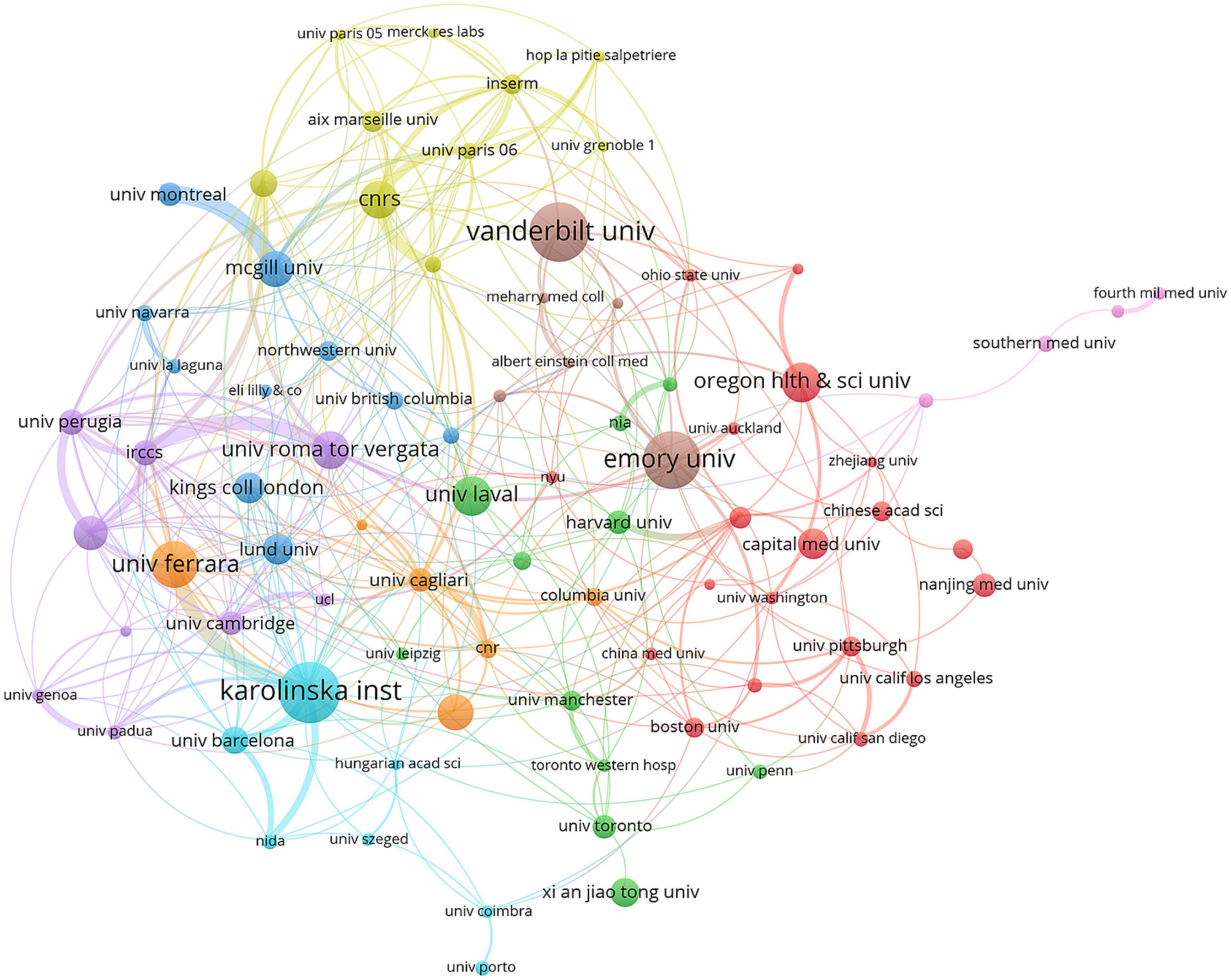
Collaboration between institutions.

Nodes in different colors represent research institutions in different countries. Università degli Studi di Ferrara and Karolinska Institute were closely linked institutions in transnational collaboration (Figure 3). Karolinska Institute (58 publications) was the most active institution, followed by Vanderbilt University (57 publications) and Emory University (54 publications) (Table 2).

**Table 2:** The top 10 productive institutions.

Rank	Institution	Documents	Citations	Average number of citations
1	Karolinska Institute	58	2,829	48.78
2	Vanderbilt University	57	4,348	76.28
3	Emory University	54	3,741	69.28
4	University of Ferrara	44	1798	40.86
5	Oregon Health and Science University	38	2096	55.16
5	Laval University	38	2041	53.71
7	CNRS	36	2,578	71.61
8	University of Rome Tor Vergata	36	2,397	66.58
9	Polish Academy of Sciences	35	1,461	41.74
10	McGill University	34	1,457	42.85

### 3.4 Journals and co-cited journals

**Figure 4:**
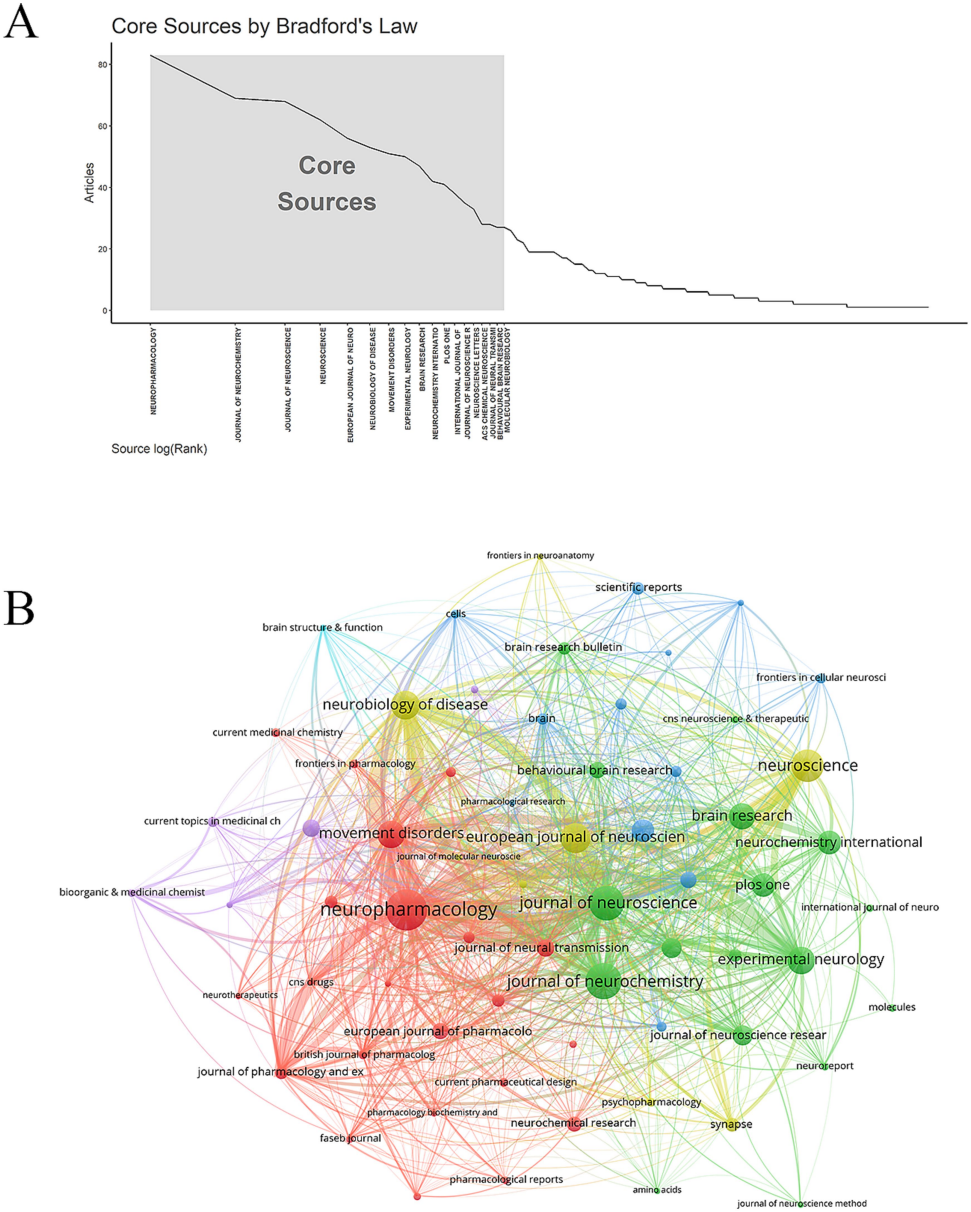
Analysis of journals. **(A)** Core Journal. **(B)** Journal network diagram.

According to Bradford’s Law (Bradford, 1934), 18 core journals related to research topics were identified (Figure 4A). These core journals were mainly related to neuroscience, covering basic research, clinical applications, and pharmacology. The Journal with the largest number of publications was *Neuropharmacology* (83 publications), followed by *Journal of Neurochemistry* (69 publications) and *Journal of Neuroscience* (68 publications) (Table 3 and Figure 4B). The co-cited journals focused on neuroscience-related research. The top three co-cited journals were *Journal of Neuroscience* (10,955 citations), *Journal of Neurochemistry* (6,151 citations), *Brain Research* (5,364 citations) (Table 4).

**Table 3:** The top 10 journals.

Rank	Source	Documents	Citations	Average number of citations	IF	JCR
1	Neuropharmacology	83	4,355	52.47	4.6	Q1
2	Journal of Neurochemistry	69	4,703	68.16	4.2	Q2
3	Journal of Neuroscience	68	7,183	105.63	4.4	Q1
4	Neuroscience	62	2,702	43.58	2.9	Q2
5	European Journal of Neuroscience	56	2,387	42.63	2.7	Q3
6	Neurobiology of Disease	53	2,151	40.58	5.1	Q1
7	Movement Disorders	51	3,323	65.16	7.4	Q1
8	Experimental Neurology	50	1757	35.14	4.6	Q1
9	Brain Research	47	1741	37.04	2.7	Q3
10	Neurochemistry International	42	1,394	33.19	4.4	Q1

IF, Impact Factor; JCR, Journal Citation Reports.

**Table 4:** The top 10 co-cited journals.

Rank	Source	Citations	IF	JCR
1	Journal of Neuroscience	10,955	4.4	Q1
2	Journal of Neurochemistry	6,151	4.2	Q2
3	Brain Research	5,364	2.7	Q3
4	Neuroscience	5,219	2.9	Q2
5	Movement Disorders	4,677	7.4	Q1
6	Proceedings of The National Academy of Sciences of The United States of America	4,352	9.4	Q1
7	Neuropharmacology	3,860	4.6	Q1
8	Journal of Biological Chemistry	3,530	4	Q2
9	Neuron	3,238	14.7	Q1
10	Neurology	3,179	8.4	Q1

IF, Impact Factor; JCR, Journal Citation Reports.

### 3.5 Authors and co-cited authors

**Figure 5:**
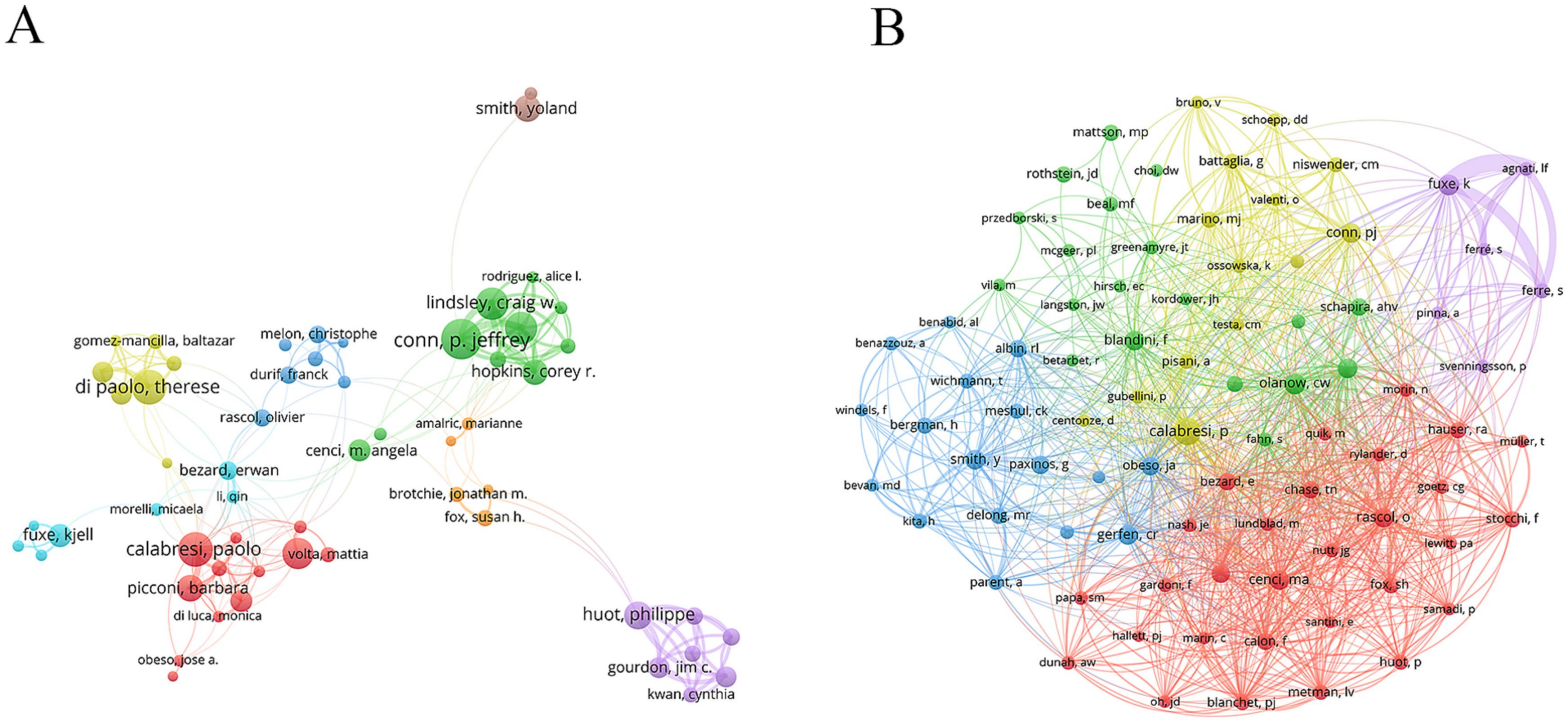
Analysis of authors and co-cited authors. **(A)** Author’s network diagram. **(B)** Co-cited author’s network diagram.

Highly productive authors are often leaders in the field of research. The author with the largest number of publications was Dr. Conn, P. Jeffrey (27 publications), followed by Dr. Di Paolo, Therese (23 publications) and Dr. Calabresi, Paolo (23 publications) (Table 5). These prolific authors were from different research teams, each with stable partners (Figure 5A). Co-cited authors reflect the relevance of the research topic or field among the authors and show the potential connection between the authors in the academic network (Figure 5B). The author with the highest co-citation was Dr. Calabresi, Paolo (615 citations), followed by Dr. Olanow, C. Warren (367 citations) and Dr. Fuxe, Kjell (348 citations) (Table 6).

**Table 5:** The top 10 authors.

Rank	Author	Documents	Citations	Average number of citations	Country	Institution
1	Dr. Conn, P. Jeffrey	27	1,258	46.59	USA	Vanderbilt University
2	Dr. Calabresi, Paolo	23	1,239	53.87	Italy	Catholic University of the Sacred Heart
2	Dr. Di Paolo, Therse	23	1,214	52.78	Canada	Laval University
4	Dr. Lindsley, Craig W.	22	828	37.64	USA	Vanderbilt University
4	Dr. Niswender, Colleen Marie	22	956	43.45	USA	Vanderbilt University
6	Dr. Liu, Jian	21	341	16.24	China	Xi’an Jiaotong University
6	Dr. Morari, Michele	21	746	35.52	Italy	University of Padua
8	Dr. Huot, Philippe	19	424	22.32	Canada	McGill University
9	Dr. Picconi, Barbara	18	1,122	62.33	Italy	IRCCS Ospedale San Raffaele
9	Dr. Smith, Yoland	18	1,090	60.56	USA	Emory University

**Table 6:** The top 10 co-cited authors.

Rank	Author	Citations	Country	Institution
1	Dr. Calabresi, Paolo	615	Italy	Catholic University of the Sacred Heart
2	Dr. Olanow, C. Warren	367	USA	Wayne State University
3	Dr. Fuxe, Kjell	348	Italy	University of Urbino
4	Dr. Blandini, F	332	Italy	University of Pavia
5	Dr. Cenci, M A	332	Sweden	University of Lund
6	Dr. Conn, P Jeffrey	329	USA	Vanderbilt University
7	Dr. Smith, Yoland	326	USA	Emory University
8	Dr. Jenner, Peter	320	UK	King’s College London
9	Dr. Gerfen, Charles R.	316	USA	National Institute of Mental Health
10	Dr. Rascol, Olivier	305	France	University of Toulouse

### 3.6 Co-cited references

**Figure 6:**
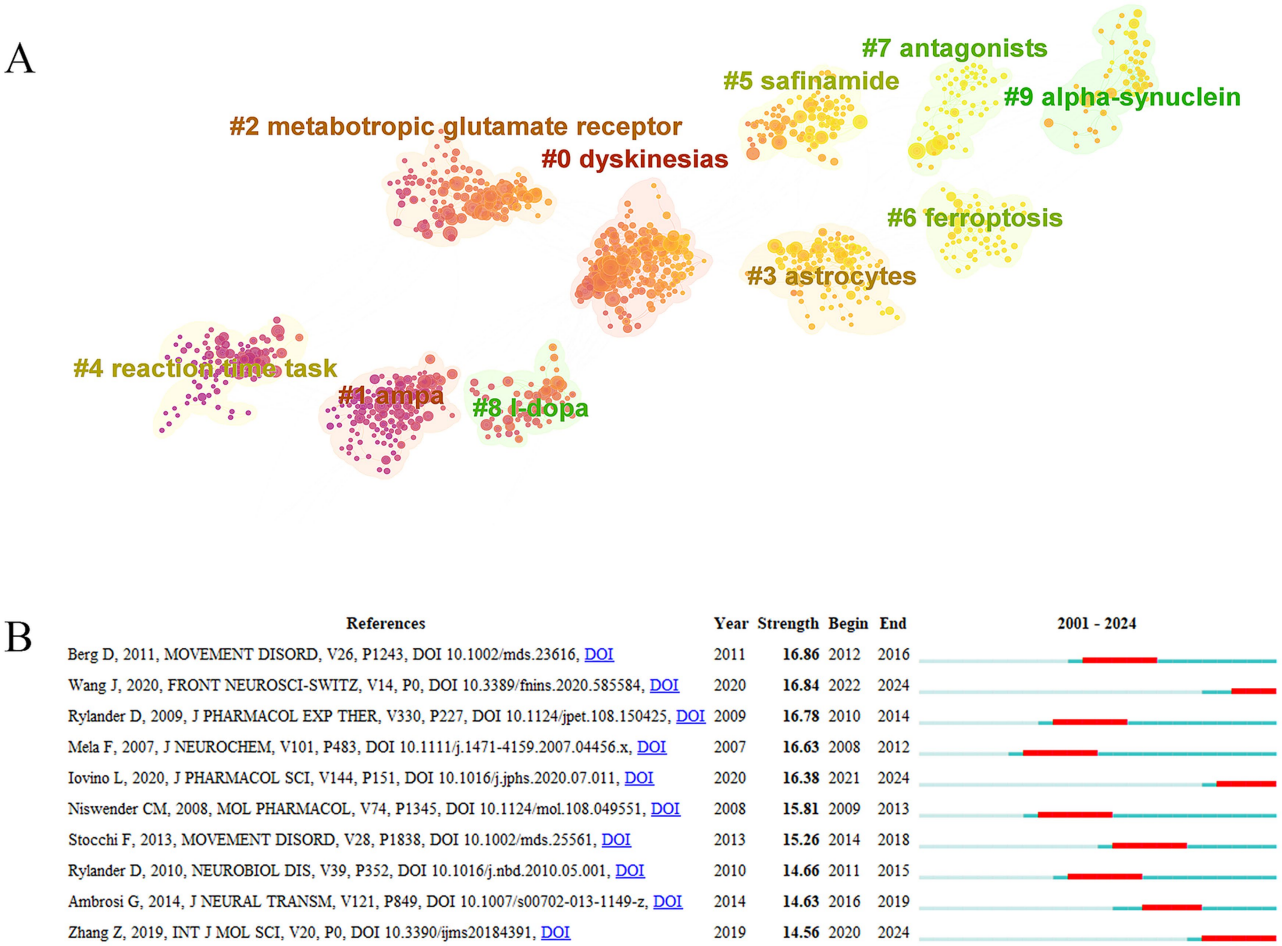
Analysis of co-cited references. **(A)** Co-cited reference cluster analysis. **(B)** Co-cited references with the strongest citation bursts.

The widely co-cited references indicate that it is an important research achievement in this field and has a major influence on related research fields. Co-cited references were ranked according to citation frequency. Table 7 listed the top 10 highly co-cited references. The paper “*The functional anatomy of basal ganglia disorders*” was the most co-cited literature (183 citations), followed by “*Primate models of movement disorders of basal ganglia origin*” (131 citations) and “*Pharmacology and functions of metabotropic glutamate receptors*” (127 citations). In cluster analysis, the modularity (Q) serves as a crucial indicator of community structure significance, with values exceeding 0.3 demonstrating robust community delineation. The silhouette (S) provides complementary validation of clustering quality, where scores above 0.7 indicate convincing and efficient partition patterns. In the reference cluster, S = 0.84 and Q = 0.93, demonstrating well-defined clusters with both strong internal cohesion and clear separation between groups. In the co-cited reference clustering, the color change from dark purple to yellow reflects the evolution of research hotspots in different periods. AMPA and metabotropic glutamate receptor have received more attention in the past; recently, the research direction has gradually shifted to astrocytes, safinamide, antagonists, ferroptosis, and alpha-synuclein (a-Syn) (Figure 6A). The co-cited reference burst can quickly identify emerging research topics that suddenly appear in a certain field. “*Molecular Mechanisms of Glutamate Toxicity in Parkinson’s Disease*,” “*Glutamate-induced excitotoxicity in Parkinson’s disease: The role of glial cells*,” and “*Roles of Glutamate Receptors in Parkinson’s Disease*” were co-cited references with strong burst power in recent years (Figure 6B).

**Table 7:** The top 10 co-cited references.

Rank	Title	Type	Citation times	Year	Journal	IF	JCR
1	The functional anatomy of basal ganglia disorders	Article	183	1989	Trends in Neurosciences	14.6	Q1
2	Primate models of movement disorders of basal ganglia origin	Article	131	1990	Trends in Neurosciences	14.6	Q1
3	Pharmacology and functions of metabotropic glutamate receptors	Article	127	1997	Annual Review of Pharmacology and Toxicology	11.2	Q1
4	Metabotropic glutamate receptors in the basal ganglia motor circuit	Review	113	2005	Nature Reviews Neuroscience	28.7	Q1
5	Loss of bidirectional striatal synaptic plasticity in L-DOPA-induced dyskinesia	Article	107	2003	Nature Neuroscience	21.3	Q1
6	L-DOPA-induced dyskinesia in the rat is associated with striatal overexpression of prodynorphin- and glutamic acid decarboxylase mRNA	Article	105	1998	European Journal of Neuroscience	2.7	Q3
7	rat brain in stereotaxic coordinates	Book	103	1998	/	/	/
8	Parkinson’s disease: Mechanisms and models	Review	100	2003	Neuron	14.7	Q1
9	Time-dependent changes in striatal glutamate synapses following a 6-hydroxydopamine lesion	Article	96	1999	Neuroscience	2.9	Q2
10	D1 and D2 dopamine receptor-regulated gene expression of striatonigral and striatopallidal neurons	Article	94	1990	Science	44.8	Q1

### 3.7 Keywords

**Figure 7:**
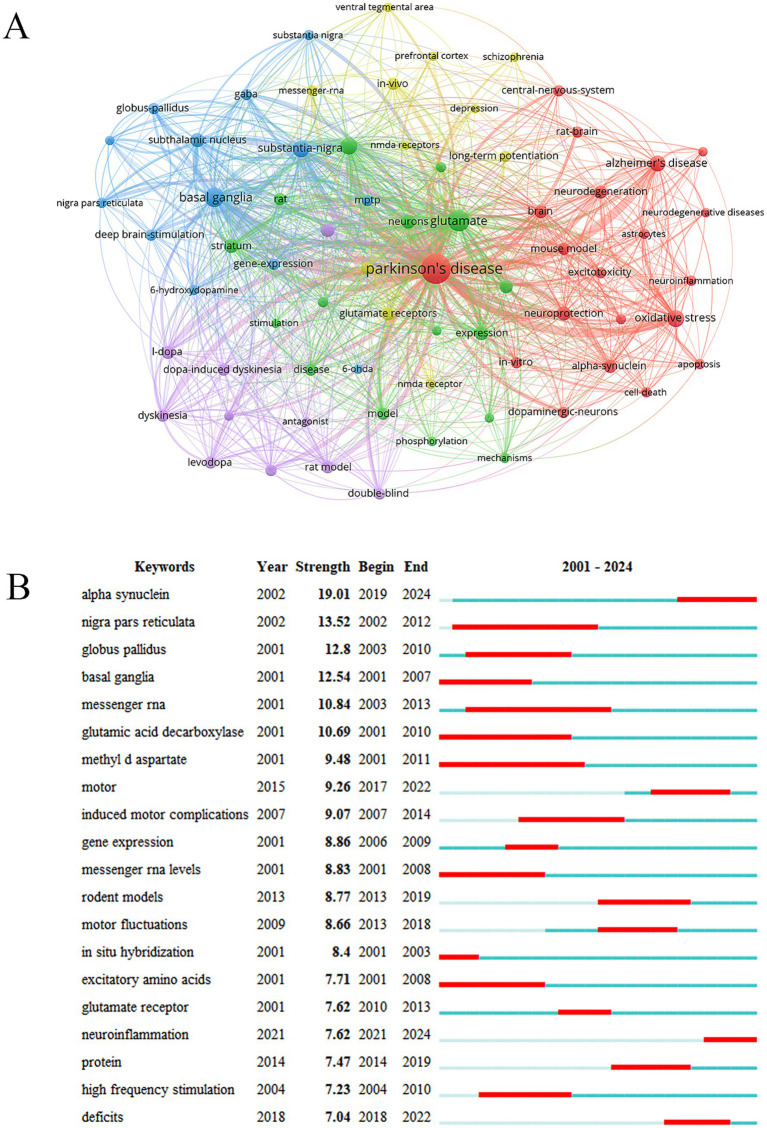
Analysis of keywords. **(A)** Keywords network diagram. **(B)** Keywords with the strongest citation bursts.

Keywords are the highly condensed and summarized core contents of the literature. High-frequency keywords often represent the research hotspots in this field. PD glutamate basal ganglia oxidative stress DA neurons a-Syn glutamate receptors and synaptic plasticity were high-frequency keywords in the research topic (Figure 7A). A keyword burst refers to a sudden increase in the frequency of a keyword at a specific time indicating that a research hotspot may be emerging. A-Syn and neuroinflammation were the keywords with strong bursts in recent years (Figure 7B).

## 4 Discussion

Glutamate, as an important excitatory neurotransmitter, is closely related to the pathogenesis and pathophysiological process of PD. This study aims to explore the hotspots and frontier trends of PD and glutamate-related research, providing an intuitive insight for researchers.

### 4.1 General information

There were 2,488 publications related to the research topic. From 2001 to 2024, the annual number of publications showed a fluctuating upward trend. The United States was a leader in this field, working closely with China, Italy, the United Kingdom, and France. Karolinska Institute was the most active institution. Core journals were mainly related to neuroscience, covering basic research, clinical applications, and pharmacology. Dr. Conn, P. Jeffrey was the most productive author. The paper “*The functional anatomy of basal ganglia disorders*” was the most co-cited literature. The emerging themes of astrocytes, ferroptosis, and a-Syn have emerged from researchers’ deepening understanding of basic science, methodological innovation, and unmet clinical needs. The central role of *α*-synuclein in the pathology of PD and the establishment of its transmission mechanism have been widely recognized. The significant role of neuroinflammation, involving astrocytes, in PD is also accepted. Ferroptosis, as a novel mechanism of cell death, aligns with the pathological features of PD. Technological breakthroughs such as high-resolution imaging/structural biology, single-cell sequencing, glial cell-specific markers/imaging, and ferroptosis-specific inhibitors/inducers and detection methods have provided key tools for in-depth research on these topics. The urgent needs for disease-modifying therapies, for improving symptom fluctuations, and for understanding new mechanisms of neuronal loss have been significant driving forces for related research. Low citation density areas included nerve growth factor (NGF), mitochondrial dysfunction, and messenger RNA (mRNA). Although neurotrophic factors have been extensively studied for dopaminergic neuron protection in PD, direct cross-disciplinary research between NGF and the glutamatergic system in PD remains relatively scarce. NGF primarily acts on cholinergic and sensory neurons, and its potential association with glutamatergic overexcitation or excitotoxicity in PD has not become a mainstream research focus. This likely contributes to the low citation density in this field, suggesting that future investigations should explore the role of NGF in regulating glutamatergic circuits during PD pathogenesis. Mitochondrial dysfunction represents a core pathological mechanism in PD, and glutamatergic excitotoxicity—mediated by excessive N-methyl-d-aspartate (NMDA) receptor activation, leading to Ca^2+^ overload—can exacerbate mitochondrial damage. Compared to research on dopaminergic neuron damage, the integration of glutamate-mediated excitotoxicity into specific molecular pathways of PD-related mitochondrial dysfunction has received less attention. The low citation density of mRNA may reflect disparities in research scope. While numerous studies on glutamate and PD focus on protein-level or functional/pharmacological mechanisms, research remains limited on dynamic changes and regulatory mechanisms of mRNA transcripts for glutamate receptor subtypes, transporters, and metabolism-related enzymes across different PD stages and brain regions. These low-citation-density domains are not insignificant; rather, they may represent emerging interdisciplinary intersections, methodological challenges, or potential knowledge gaps within the current glutamate-PD research network.

### 4.2 Hotspots and frontiers

High-frequency keywords often represent the research hotspots in this field. PD, glutamate, basal ganglia, DA, oxidative stress, a-Syn, glutamate receptors, and synaptic plasticity were high-frequency keywords in the research topic. Glutamate plays a key role in basal ganglia circuits. With nigrostriatal dopaminergic depletion, glutamatergic projections from the subthalamic nucleus to the output nuclei of the basal ganglia become hyperactive, and glutamate receptor regulation changes (Blandini et al., 1996). Glutamate receptor-mediated excitotoxicity is a key mechanism of neuronal death in PD (Ambrosi et al., 2014). Glutamate receptors affect motor control by regulating synaptic plasticity in the basal ganglia (Berretta et al., 2008) and are also involved in learning and memory processes (Riedel et al., 2003). Glutamate receptors are mainly classified into ionotropic glutamate receptors (iGluRs) and mGluRs. iGluRs can be further divided into NMDA receptors, *α*-amino-3-hydroxy-5-methyl-4-isoxazolepropionic acid (AMPA) receptors, and kainate receptors while mGluRs can be further divided into eight receptor subtypes, from mGluRs1 to mGliRs8 (Wang et al., 2020). Long-term enhancement (LTP) and long-term inhibition (LTD) are the two main forms of synaptic plasticity. During LTP induction, a large amount of glutamate is released and binds to AMPA receptors on the postsynaptic membrane, increasing the sensitivity of the postsynaptic membrane to glutamate, thereby enhancing synaptic transmission and achieving synaptic plasticity (Collingridge and Abraham, 2022). Activated mGluRs initiate a series of intracellular signal transduction events that ultimately lead to a long-term reduction in synaptic transmission efficacy, forming LTD (Frenguelli, 2013). mGluR5 specifically activates TRPC1 channels to participate in synaptic plasticity and memory formation (Lepannetier et al., 2018).

Keyword bursts can quickly capture research topics that suddenly receive much attention at a specific time representing rapidly developing areas with great research potential. a-Syn and neuroinflammation were the keywords with strong bursts in recent years. The accumulation of protein aggregates formed by a-Syn in the nervous system is one of the pathological hallmarks of PD (Stefanis, 2012). a-Syn promotes the release of glutamate and glutamate excitotoxicity aggravates a-Syn aggregation. a-Syn oligomers increase abnormal glutamate release by disrupting synaptic vesicle recycling

which leads to hyperactivation of glutamatergic signaling (Danzer et al., 2007). Glutamate leads to intracellular calcium overload and calcium-dependent protease activation through NMDA receptor activation which promotes abnormal phosphorylation and aggregation of a-Syn forming a positive feedback loop (Fujiwara et al., 2002). In PD abnormal aggregation of a-Syn or neuronal injury can activate microglia to release pro-inflammatory factors such as TNF-*α* and IL-1β (Béraud et al., 2013). In neuroinflammation activated microglia interact with reactive astrocytes

leading to increased extracellular glutamate concentrations (Takaki et al., 2012). Glutamate accumulated at synapses binds to glutamatergic receptors on microglia and aggravates neuroinflammation (Haroon et al., 2017).

### 4.3 Drug clinical research

Current pharmacological interventions focusing on glutamate receptor modulation include AMPA receptor antagonists, NMDA receptor antagonists, and mGluR5 antagonists. Two phase III clinical trials evaluating the AMPA antagonist perampanel failed to significantly alleviate motor symptoms in PD patients (Lees et al., 2012). A randomized controlled trial found that the NMDA receptor antagonist amantadine exhibited a clinically significant reduction in dyskinesia and reduced off-time in PD patients (Oertel et al., 2017). Two phase 2 randomized, double-blind studies showed that the mGluR5 inhibitor mavoglurant did not achieve alleviation in levodopa-induced dyskinesia (LID) in PD (Negida et al., 2021). A phase IIA clinical trial showed that the mGluR5 inhibitor dipraglurant was safe and well tolerated when first administered to PD patients, but further study with more patients is needed to alleviate motor deficits (Tison et al., 2016).

Strategies to target a-Syn include immunotherapy and small molecule inhibitors. PRX002, an anti-a-Syn monoclonal antibody, has been shown to reduce plasma a-Syn levels in PD patients in phase II clinical trials (Jankovic et al., 2018). In animal experiments, a-Syn aggregation inhibitor anle138b could inhibit the disease progression in PD mice (Levin et al., 2014). A clinical study showed that the anle138b had a good safety profile in healthy human volunteers, but further clinical trials in PD patients are needed (Levin et al., 2022). Drugs targeting neuroinflammation include the minocycline and pioglitazone. Minocycline did not show safety concerns in phase II clinical trials, but its reduced tolerability is also a concern (NINDS NET-PD Investigators, 2008). PPAR-*γ* agonist pioglitazone can inhibit the activation of microglia and has a neuroprotective effect. A phase II study found that pioglitazone was less effective as an intervention to slow early PD progression, and large-scale further studies were not recommended (NINDS Exploratory Trials in Parkinson Disease (NET-PD) FS-ZONE Investigators, 2015).

### 4.4 Challenge and opportunity

The development of disease-modifying therapies for PD remains constrained by the limited availability of clinically validated drugs, the long time to develop drugs, and rigorous safety analyses. Although NMDA receptor antagonists are effective against symptoms in the treatment of LID, challenges remain in mitigating adverse effects. *α*-Syn-targeted strategies face dual hurdles: precise differentiation between pathological aggregates and physiological monomers and overcoming the inefficiency of therapeutic biologics in penetrating the blood–brain barrier may require advanced delivery platforms such as lipid nanoparticles. The pathophysiological interplay between neuroinflammatory cascades (such as microglial activation and cytokine release) and glutamatergic dysregulation creates a self-reinforcing cycle that limits the efficacy of a single target. Furthermore, anti-inflammatory therapy treatment may lead to immunosuppression or metabolic perturbations, demanding careful risk–benefit analysis in PD patients. Emerging glutamate-related biomarkers including cerebrospinal fluid glutamate elevation, reduced excitatory amino acid transporter 2 expression, and striatal glutamate-glutamine complex signals detected by proton magnetic resonance spectroscopy exhibit promise for early diagnosis and therapeutic stratification. However, standardization and clinical validation remain prerequisites for routine implementation. Potential future directions and opportunities are as follows: (i) The clinical application of glutamate-related markers can be promoted. (ii) Development of nano delivery systems to improve central drug permeability. (iii) Further optimization of multi-target drug strategies, multi-drug approaches simultaneously targeting glutamatergic signaling, α-Syn pathology, and neuroinflammation. (iv) Precision editing to silence pathogenic variants or up-regulate neuroprotective pathways in combination with gene editing and other technologies.

### 4.5 Limitations

This study also has some limitations. First, the data were sourced exclusively from WoS and do not include literature from other databases. Although widely recognized by researchers, the WoS database cannot cover all relevant publications in the field and thus may miss significant works indexed elsewhere. Second, co-citation analysis predominantly identifies classic papers with long citation histories. Consequently, newly published, high-impact studies (even critically important ones) may be underrepresented or overlooked due to insufficient time to accumulate co-citations. This time lag can hinder the timely identification of current research frontiers. Furthermore, highly cited papers are more likely to receive further citations (including co-citations), making established influential authors/research more visible while potentially obscuring high-quality work in emerging fields or by independent researchers. Third, citation frequency is not a sole indicator of scientific quality. Citations can be influenced by factors such as journal impact factor/prestige, author/institutional reputation, and language or geographical biases. Fourth, while burst keywords signal a sharp increase in usage frequency, their causes are multifaceted. They might indicate genuine emerging hotspots, but could also stem from terminology shifts, the publication of highly cited reviews, concentrated output by specific groups, or intense debate surrounding specific findings. Despite these limitations, bibliometric methods provide a valuable, macro-level, and data-driven comprehensive overview of the domain structure.

## 5 Conclusion

Research on PD and glutamate focused on countries with increasing aging. The collaboration of different countries and institutions was conducive to promoting the development of this field. The research hotspots included basal ganglia, oxidative stress, dopamine, neurons, a-Syn, glutamate receptors, and synaptic plasticity. a-Syn and neuroinflammation may be research directions in the future.

## Data Availability

The original contributions presented in the study are included in the article/supplementary material, further inquiries can be directed to the corresponding authors.
